# The Rotational Barrier in Ethane: A Molecular Orbital Study

**DOI:** 10.3390/molecules17044661

**Published:** 2012-04-20

**Authors:** Ramiro F. Quijano-Quiñones, Mariana Quesadas-Rojas, Gabriel Cuevas, Gonzalo J. Mena-Rejón

**Affiliations:** 1 Laboratory of Pharmaceutical Chemistry, Faculty of Chemistry, Autonomous University of Yucatan, 41 No. 421 Col. Industrial, C.P. 97150, Merida, Yucatan, Mexico; Email: mariana_quesadas@yahoo.com.mx (M.Q.-R.); mrejon@uady.mx (G.J.M.-R.); 2 Institute of Chemistry, National Autonomous University of Mexico, Circuito Exterior, Ciudad Universitaria, C.P. 04510 Mexico D.F., Mexico; Email: gecgb@unam.mx

**Keywords:** staggered conformation, ethane, rotational barrier, molecular orbital, DFT

## Abstract

The energy change on each Occupied Molecular Orbital as a function of rotation about the C-C bond in ethane was studied using the B3LYP, mPWB95 functional and MP2 methods with different basis sets. Also, the effect of the ZPE on rotational barrier was analyzed. We have found that σ and π energies contribution stabilize a staggered conformation. The σ_s_ molecular orbital stabilizes the staggered conformation while the 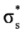 stabilizes the eclipsed conformation and destabilize the staggered conformation. The π_z_ and 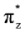 molecular orbitals stabilize both the eclipsed and staggered conformations, which are destabilized by the π_v_ and 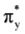 molecular orbitals. The results show that the method of calculation has the effect of changing the behavior of the energy change in each Occupied Molecular Orbital energy as a function of the angle of rotation about the C–C bond in ethane. Finally, we found that if the molecular orbital energy contribution is deleted from the rotational energy, an inversion in conformational preference occurs.

## 1. Introduction

The existence of a rotational barrier of 2.875 kcal·mol^−1^ about the C-C bond in ethane has been known for many years [1,2,3,4,5,6,7,8,9]. There are two main effects that have been regarded as responsible for this rotational barrier: A steric repulsion in the eclipsed conformation [2,3,4,9,10,11,12,13,14] and an enhanced stabilization of the staggered conformation due to hyperconjugation [5,8,15,16,17,18,19,20,21,22]. 

The steric effect has its origin in the fact that atoms in molecules occupy a certain amount of space, resulting in changes in shape, energy, and reactivity. It is an essential concept in chemistry, biochemistry, and pharmacology, influencing rates and energies of chemical reactions, impacting structure, dynamics, and function of enzymes, and to a degree, governing how and at what rate a drug molecule interacts with a receptor. Different approaches have been proposed to quantify the steric effect. For example, Shubin Liu recently proposed an energy partition scheme under the framework of Density Functional Theory (DFT) [23,24]. In this scheme the total energy density functional is decomposed into three independent contributions from steric, electrostatic, and quantum effects. This scheme was used to explore the internal rotation barrier of various molecules [14,25,26,27]. In particular, they analyzed the origin of the rotation barrier in ethane and concluded that the eclipse conformer possesses a large steric repulsion than the staggered conformer [14] in support of the steric repulsion as the cause for the preferred staggered conformation of ethane. However, to date there is no consensus about which is the origin of the steric effect and about the method to use to quantify it. For example, Weisskopf [28] attributed it to the “kinetic energy pressure” in atoms and molecules, whereas others [9,10,12,13,29] employed the quantum contribution from the Pauli Exclusion Principle [30,31,32,33,34]. Therefore, due to the different approximations used for the calculation of steric effect it is not possible to draw final conclusions from these calculations. Nevertheless, the steric repulsion still remains the most popular explanation for the preferred staggered conformation of the ethane [2,3,4,9,10,11,12,13,14].

Alternatively, the possible role of hyperconjugation effect in the ethane rotation barrier has been conjectured for many years [21,22]. Hyperconjugation corresponds to the interaction between an occupied bond orbital and a vicinal unoccupied antibond orbital, which results in an occupied delocalized orbital and in stabilization of the system. Lately, Pophristic and Goodman [15] have argued that the rotational energy barrier cannot be explained by steric repulsion between vicinal C–H bonds in the eclipsed conformation. They proposed that the staggered conformation results from hyperconjugation effects. In this conformation, the C–H bonds have a favorable disposition for the interaction of the σ^*^ antibonding orbital of one C–H unit with the corresponding occupied σ bonding orbital at the other side. They have used a natural bond orbital (NBO) [35], analysis to prove the existence of hyperconjugation effects by deleting the σ–σ^*^ interactions. The conclusion of this method is supported by other similar studies [16,17,18,19,20]. Nevertheless, the NBO analysis for the ethane conformations has been challenged by different authors because NBO does not leave the electron density and the energy unchanged, thus causing an energy lowering that affects the final result [10,36].

Therefore, there is no definite explanation for the driving force of the preferred ethane conformation mainly due to the different approximations used for the calculation of steric and hyperconjugative effects, in addition to the difficulty of their simultaneous calculation and because hyperconjugation, steric repulsion, and possibly some other effects coexist entangled in the ethane molecule. In consequence, different authors obtain different amounts of steric and hyperconjugation effects. 

In order to contribute to the understanding of the conformational driving force in ethane, we propose an alternative point of view based on a systematic analysis of its Molecular Orbitals (MOs), the most basic concept in conformation, to assign the different MOs to each of the preferred conformations and estimate the overall net effect by subtracting the molecular orbital energy from the total energy during the rotation about the C–C bond in the ethane. In addition, we propose to study the effect that this theoretical model has on the behavior of energy of the MOs as a function of angle of rotation about C–C bond in ethane.

Based on these considerations, we carried out the analysis of electronic and structural properties of ethane as a function of the C–C angle (φ) rotation. The DFT (B3LYP, mPWB95) and MP2 methods with 6-31G(d, p), 6-31+G(d, p), and 6-31++G(d, p) basis sets were used to evaluate the effects of these models in the ethane rotational molecular orbital energy and the relationship to the ethane-preferred conformation. The B3LYP functional was used because of its wide application to calculate electronic structure, reaction and activation energies. However, there is evidence that the B3LYP method usually underestimates barrier heights [37,38]. Additionally, the third generation mPWB95 functional was applied since recent studies in small systems have shown that it yields more reliable results than the B3LYP functional [39,40]. We compared the results calculated with DFT and those calculated with MP2 theory since it its known that B3LYP and MP2 give errors in opposite direction in the energy for organic molecules [41]. In addition, we compared the basis 6-31G(d,p), 6-31+G(d,p), and 6-31++G(d,p) in order to evaluate the effect of the addition of polarization and diffusion to the basis set. Finally, in order to evaluate the effect of zero-point energy (ZPE) on rotational barrier, the calculations were performed with- or without consideration of ZPE. All energies reported with zero-point corrections are not scaled for comparative purpose.

The aim of this work is to contribute to understanding of the contribution of each of the molecular orbitals in ethane to the rotational barrier and its overall net effect. In addition, we propose analyze the effect that theoretical model has on the behavior of energy of the MOs as a function of angle of rotation about C–C bond in ethane. To the best of our knowledge, this is the first study of energy changes on each orbital in ethane by different methods.

## 2. Results and Discussion

The Kohn-Sham total energy rotation (E_rot_) for conversion of ethane from staggered to eclipsed conformation was calculated in the gas phase and the geometries were fully optimized. In the applied models the total energy of ethane is calculated as a function of the torsion angle φ, obtaining an energy minimum at the staggered (E_s_) conformation and a maximum at the eclipsed (E_e_) conformation. The energy difference between E_e_ and E_s_ (E_e_−E_s_) is the calculated rotational barrier (ΔE_rot_).

Regarding evaluation of the of ZPE effect on the rotational barrier it is important to notice that if ZPE is not included (Table 1), the MP2 model seems to overestimate the rotational barrier while the DFT model underestimates it. In addition, the inclusion of diffuse functions provokes a decrease of the rotational barrier. Finally, the B3LYP/6-31G(d,p) level of theory allowed to obtain the closest rotational barrier (2.8020 kcal·mol^−1^) to the experimental value (2.875 kcal·mol^−1^). Similar results are observed when ZPE was included for MP2/6-31G(d, p) level of theory, which estimates a rotational barrier of 2.9116 kcal·mol^−1^. 

**Table 1:** Calculated values of ΔE_rot_ (kcal·mol^−1^).

	B3G	B3+G	B3++G	MPG	MP+G	MP++G	MP2G	MP2+G	MP2++G
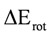	2.803	2.732	2.736	2.752	2.673	2.683	3.025	2.966	2.981
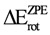	2.541	2.466	2.472	2.504	2.422	2.435	2.912	2.799	2.761

Labels B3, MP correspond to the B3LYP and mPWB95 functionals, MP2 correspond to the Moller-Plesset perturbation theory of order 2 and the symbols G, +G, and ++G represent the basis set 6-31G(d, p), 6-31+G(d, p), and 6-31++G(d, p), respectively.

The lengths of the C–C and C–H bonds were obtained as a function of φ. It is interesting to note that when the conformation goes from eclipsed (φ = 0°) to staggered (φ = 60°) the C–C bond length decreases slightly, while the C–H bonds increase slightly. The percent decrease of C–C bond length varies from 0.9 when the calculation is performed with B3LYP/6-31G(d,p) to 0.85 if the MP2/6-31G(d,p) method is applied. For the C–H bond length increase the percent variation is from 0.089 [MP2/6-31G(d,p)) to 0.1 (B3LYP/6-31G(d,p)]. Thus, rotation about the C–C bond generates a small effect over the geometry of ethane.

**Figure 1:**
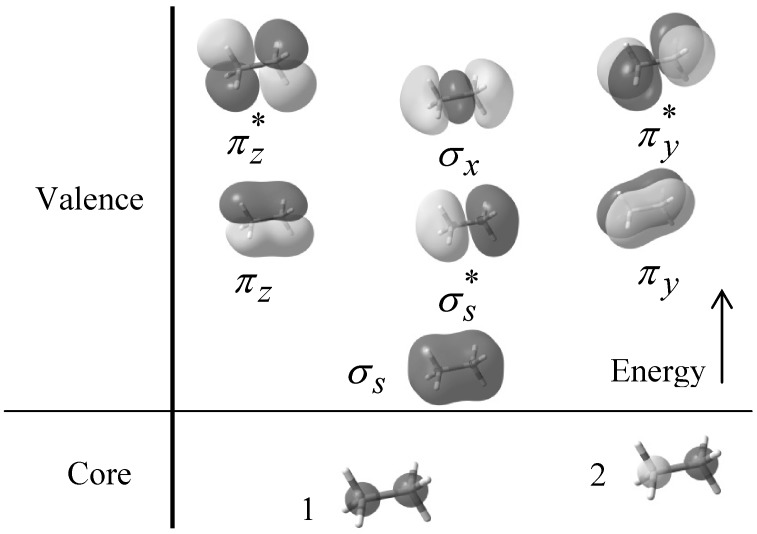
Filled molecular orbitals of ethane calculated with B3LYP/6-31+G(d, p) theoretical model.

Ethane consists of two carbon and six hydrogen atoms sharing nine filled MOs. The valence molecular orbital configuration appropriate to the D_3d_ symmetry staggered conformation is *(2a_1g_)^2^(2a_2u_)^2^(e_g_)^4^(3a_2u_)^2^(e_u_)*^4^ while that of the D_3h_ symmetry of the eclipsed conformer is *(2a´_1_)^2^(2a´´_2_)^2^(e´´)^4^(3a´´_2_)^2^(e´)^4^*. The contributions from “*a*” category orbitals represent the *s* orbital interactions and those from “*e*” category orbitals represent *p* orbital interactions [15,42]. For this reason the MOs are called σ_s_, 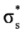, π_v_, π_z_, σ_x_, 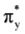, and 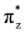 respectively. Figure 1 also shows the core orbital´s and the 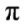 character of the degenerate π_v_, π_z_, 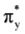, and 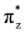 molecular orbitals, and the σ character of the σ_s_, 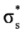 and σ_x_ molecular orbitals at B3LYP/6-31+G(d, p) level of theory. 

**Figure 2:**
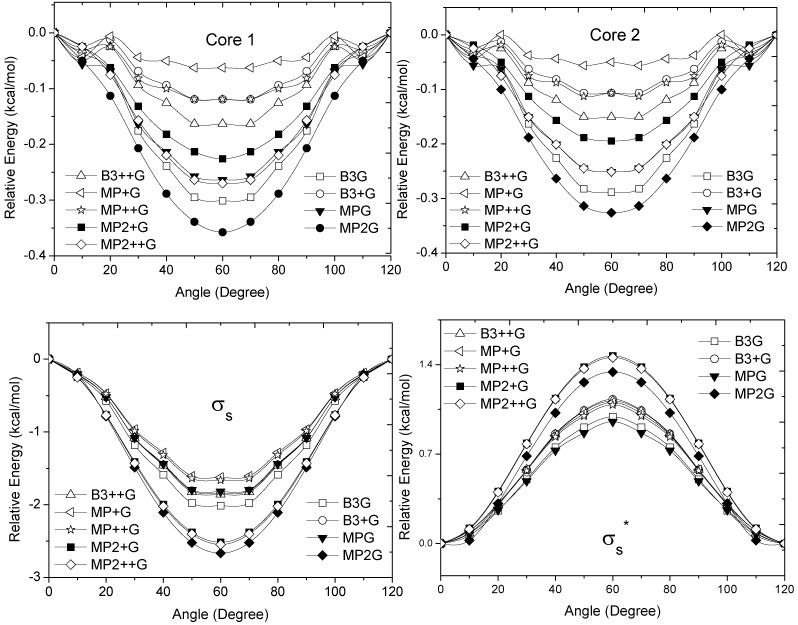
Energy of the core, 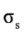 and 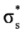 occupy MOs of ethane as a function of the rotational angle, calculated with the entire theoretical model studied in this work. Labels B3, MP correspond to the B3LYP and mPWB95 functionals, MP2 correspond to HF theory and the symbols G, +G, and ++G represent the basis set 6-31G(d, p), 6-31+G(d, p), and 6-31++G(d, p), respectively.

**Figure 3:**
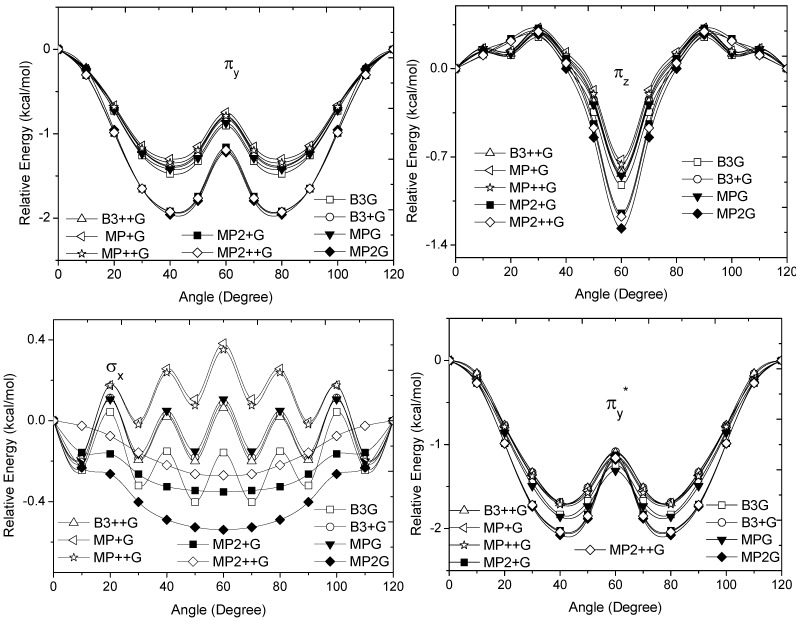
Energy of the π_v_, π_z_, σ_x_ and 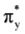 occupy MOs of ethane as a function of the rotational angle, calculated with the entire theoretical model studied in this work. Labels B3, MP correspond to the B3LYP, and mPWB95 functionals, MP2 correspond to the HF theory and the symbols G, +G and ++G represent the basis set 6-31G(d, p), 6-31+G(d, p), and 6-31++G(d, p), respectively.

**Figure 4:**
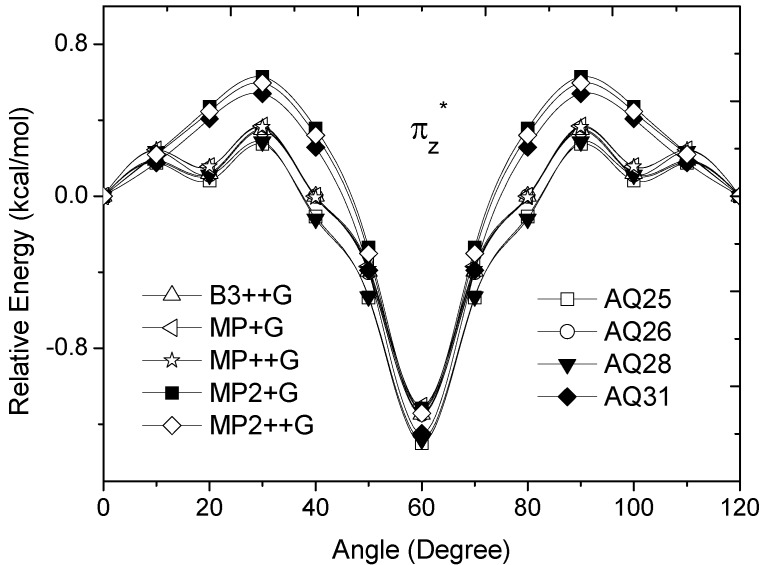
Energy of the 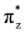 occupied MO of ethane as a function of the rotational angle, calculated with the entire theoretical model studied in this work. Labels B3, MP correspond to the B3LYP and mPWB95 functionals, MP2 correspond to the HF theory and the symbols G, +G, and ++G represent the basis set 6-31G(d, p), 6-31+G(d, p), and 6-31++G(d, p), respectively.

Along the potential energy surface (PES) the MOs are changing, but, for simplicity we retain the labels of the MOs in its evolution as a function of angle of rotation. The energy changes in the MOs, calculated as a function of φ are shown in Figure 2, Figure 3 and Figure 4. Since there are no MOs associated to the MP2 energies, we plotted the energy of the canonical Hartree-Fock (HF) MOs associated to each MP2. Each MO core showed a minimum energy at the staggered conformation (Figure 2). The hydrogen atoms have MOs with large *s *character. The 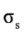 and 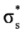 orbitals present mainly C(1*s*) and C(2*p*) character and are main contributors to the C–C bond strength. All the 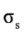 bonding orbitals exhibited a minimum energy at staggered conformation (Figure 2). It is possible to observe that the energy changes between eclipsed and staggered conformations fluctuate from 2.667 kcal·mol^−1^ (HF/6-31G(d,p)) down to 1.626 kcal·mol^−1^ (mPWB95/6-31+G(d,p)).

These results show the importance of the 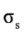 orbital bonding for the rotational barrier. Interestingly, the 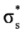 antibonding molecular orbital presented a minimum energy at the eclipsed conformation and a maximum at the staggered conformation with energies that change from −1.4683 kcal·mol^−1^ [HF/6-31G(d,p)] down to −0.9538 kcal·mol^−1^ [mPWB95/6-31+G(d,p)]. The negative sign of the value reveals that the 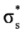 molecular orbital had a minimum energy at φ = 0°, which stabilized the eclipsed conformer.

The bonding π_v_ and π_z_, and antibonding 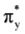 and 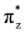 sets have a large H(1*s*) character and C(2*p*) character. These MOs are mainly associated to the vicinal hyperconjugative delocalization interactions between the methyl groups. The energy values, calculated for the change from staggered to eclipsed conformation in the orbitals π_z_, π_v_, 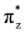 and 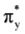, vary from 0.7216 [mPWB95/6-31G(d,p)], 0.7467 [mPWB95/6-31+G(d,p)], 1.1044 [mPWB95/6-31+G(d,p)] and 1.1546 kcal·mol^−1^ [mPWB95/6-31+G(d,p)] to 1.267 [HF/6-31G(d,p)], 1.794 [HF/6-31G(d,p)], 1.2989 [B3LYP/6-31G(d,p)] and 1.3114 kcal·mol^−1^ [B3LYP/6-31+G(d,p)], respectively (Figure 3 and Figure 4).

The calculated energy change for the studied MOs was equivalent to ΔE_rot_, demonstrating the role of these orbitals for the rotational barrier of ethane. However, for all models, the bonding and antibonding π_v_ and 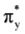 orbitals showed a smaller local maximum energy at staggered rather than at eclipsed conformation. Additionally, the energy presented a symmetric double minimum structure close to φ = 40° and φ = 80°. These results demonstrate that the energy changes in the π_v_ and 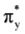 MOs destabilize the staggered conformer.

The total energy of the π_z_ and 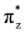 MOs showed an asymmetric double minimum structure at φ = 0° (eclipsed conformation; local minimum) and φ = 60° (staggered conformation; global minimum) in addition to two maxima close to φ = 20° and φ = 100°. Thus, the energy change in the π_z_ and 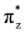 MOs stabilizes both conformations. It is important to remark, however, that calculations based on DFT provided two energy minima close to φ = 40 and φ = 100°, which were not found when the HF model was used.

Finally, the molecular orbital σ_x_, exhibited large H(1*s*) and C(2*p*) characters. The calculated values for the change between eclipsed and staggered conformations, showed variations from −0.3827 kcal·mol^−1^ [HF/6-31G(d,p)] to 0.5396 kcal·mol^−1^ [mPWB95/6-31G(d,p)] (Figure 3). In this case, calculations was carried out using the HF model showed an energy minimum at φ = 60°; revealing that this MO contributes to stabilize the staggered conformation. On the other hand, calculations based on DFT indicated irregular changes in energy, while only the B3LYP/6-31G(d,p) model predicts that the staggered conformation is stabilized by the contribution of the σ_x_ orbital.

From Figure 3 and Figure 4, we can see that the DFT and HF methods predict different behaviors in the molecular orbital energy of the σ_x_, π_z_, and 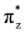 MOs. The total electronic energy of the molecular orbital 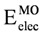 was calculated as a function of φ, considering two electrons for each MO. In all cases, the minimum and maximum values of both 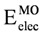 and 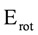 coincided. The overall net effect of the 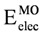 in conformational preference of the ethane can be estimated if the difference in energy between the 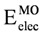 in the eclipsed conformation and 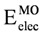 in staggered conformation (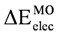) is compared to 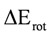. We can see from the Table 2 that the 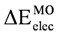 was higher than the 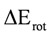 for all methods and basis set, in spite of the difference found between the different models in the behavior of the MO as a function of φ. This indicates that the energy difference between 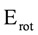 and 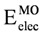 as a function of φ produced an inversion of the minimum. Accordingly if 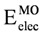 is subtracted, the preference of 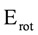 for the staggered conformer is lost, and the eclipsed conformer becomes more stable. Additionally, we can note that the calculations using the HF model predict a higher 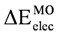 than those performed with DFT, while the use of the functional mPWB95 of DFT allowed predict a lower value of 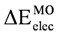 (Table 2). Finally, the inclusion of diffuse functions in all calculations resulted in a decrease of 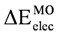.

**Table 2:** Calculated values of 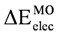, σ (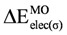) and π (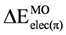) contributions compared to ΔE_rot_ and 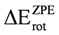 (Kcal/mol).

	B3G	B3+G	B3++G	MPG	MP+G	MP++G	MP2G	MP2+G	MP2++G
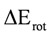	2.803	2.732	2.736	2.752	2.673	2.683	3.025	2.966	2.981
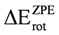	2.541	2.466	2.472	2.504	2.422	2.435	2.912	2.799	2.761
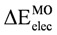	12.27	9.400	9.79	11.170	7.919	8.559	14.92	12.75	13.29
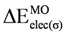	3.539	1.694	1.983	2.548	0.502	0.904	5.095	3.640	3.953
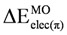	8.735	7.706	7.806	8.622	7.417	7.656	9.827	9.111	9.337

Labels B3, MP correspond to the B3LYP and mPWB95 functionals, MP2 correspond to the HF theory and the symbols G, +G, and ++G represent the basis set 6-31G(d, p), 6-31+G(d, p), and 6-31++G(d, p), respectively.

The value of total σ (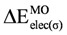) and π (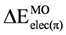) contributions to 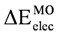 for all used models is shown in Table 2. It was found that when the σ contribution is deleted, the conformational preference is reversed. A similar behavior was observed for the total π energy contribution to 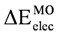. It is important to note that the π energy contribution is higher than the σ contribution.

## 3. Experimental

The quantum chemical calculation was performed using the GAUSSIAN 09 code [43] The total energy dependence in the torsional angle was calculated using Density Functional Theory (DFT) [44], with a B3LYP, mPWB95 functional and MP2 method [45], and 6-31G(d, p), 6-31+G(d, p) and 6-31++G(d, p) basis set. The rotation about the central carbon-carbon single bond from eclipsed to staggered conformation was considered at 10° intervals. Full optimization of C–C bond and CH_3_ geometries of the ethane were carried out in all calculations.

## 4. Conclusions

The calculated rotational energy barriers at different levels show that it is not necessary to incorporate diffusion functions for an accurate description of the energetic barrier in ethane. It is important to note that the B3LYP/6-31G(d,p) model underestimates the value of the rotational barrier in ethane, while the MP2/6-31G(d,p) model overestimates it. In addition, the functional mPWB95 predict the worst values for rotational barrier and MP2/6-31+G(d, p) predicts the higher energy changes. We have found that the π_v_ and 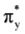 orbitals showed a smaller local maximum energy at staggered than at eclipsed conformation. In addition, the energy change of the π_z_ and 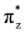 MO’s stabilizes the eclipsed and the staggered conformations. The DFT methods predict two energy minima close to φ = 40 and φ = 100°. The π_z_ and 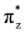 orbitals stabilize both conformations. For the σ_x_ MO, the DFT energy changes contribute to stabilize the staggered conformation and shows irregular behavior. In addition, we found that for all models if 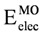 is subtracted from the total energy of the ethane, the conformational preference in ethane is the eclipsed conformer.
